# The structural response of the cornea to changes in stromal hydration

**DOI:** 10.1098/rsif.2017.0062

**Published:** 2017-06-07

**Authors:** Sally Hayes, Tomas White, Craig Boote, Christina S. Kamma-Lorger, James Bell, Thomas Sorenson, Nick Terrill, Olga Shebanova, Keith M. Meek

**Affiliations:** 1 Structural Biophysics Group, School of Optometry and Vision Sciences, Cardiff University, Cardiff, UK; 2 ALBA Synchrotron Light Source, Cerdanyola del Vallès, Barcelona, Spain; 3 Diamond Light Source, Didcot, Oxfordshire, UK

**Keywords:** cornea, swelling, hydration, collagen, proteoglycans, fixed charge

## Abstract

The primary aim of this study was to quantify the relationship between corneal structure and hydration in humans and pigs. X-ray scattering data were collected from human and porcine corneas equilibrated with polyethylene glycol (PEG) to varying levels of hydration, to obtain measurements of collagen fibril diameter, interfibrillar spacing (IFS) and intermolecular spacing. Both species showed a strong positive linear correlation between hydration and IFS^2^ and a nonlinear, bi-phasic relationship between hydration and fibril diameter, whereby fibril diameter increased up to approximately physiological hydration, *H* = 3.0, with little change thereafter. Above *H* = 3.0, porcine corneas exhibited a larger fibril diameter than human corneas (*p* < 0.001). Intermolecular spacing also varied with hydration in a bi-phasic manner but reached a maximum value at a lower hydration (*H* = 1.5) than fibril diameter. Human corneas displayed a higher intermolecular spacing than porcine corneas at all hydrations (*p* < 0.0001). Human and porcine corneas required a similar PEG concentration to reach physiological hydration, suggesting that the total fixed charge that gives rise to the swelling pressure is the same. The difference in their structural responses to hydration can be explained by variations in molecular cross-linking and intra/interfibrillar water partitioning.

## 1. Background

The outer covering of the eye comprises a strong, transparent cornea and an opaque sclera. The transparency and precise shape of the cornea (in the central region in particular [1]) are essential to its function as they enable it to focus and transmit almost all incident light in the visible spectrum onto the lens and retina. The cornea comprises several layers, the largest being the stroma, which occupies approximately 90% of the total corneal thickness and is composed chiefly of water, collagen, proteoglycans and keratocytes. The hydration (*H*) of the cornea, defined as the ratio of the weight of water to the dry weight, is close to *H* = 3.2 for most species at physiological levels [2].

Within the stroma, thin collagen fibrils lie parallel to each other within stacked layers (lamellae) which are themselves interspersed with thin, flat keratocytes. Although most lamellae lie parallel to the corneal surface [3], lamellar interweaving is a common feature of the anterior [4] and mid-stroma [5]. The small diameter of the collagen fibrils and their regular separation distance, when observed at physiological hydration (*H*_phys_), are believed to be regulated by the charge density on the proteoglycans [6–9]. As detailed in a review article by Meek & Knupp [10], the transparency of the cornea at *H*_phys_ can be primarily attributed to the specific arrangement of collagen fibrils within the corneal stroma and to the refractive index of its constituent cells [11]. However, damage to the corneal endothelial or epithelial cell layers can result in stromal oedema (predominantly in the posterior third of the cornea [12,13]) and a significant loss of transparency. The increased light scatter that occurs when the tissue swells is believed to be the result of a non-uniform distribution of water and a disruption to stromal collagen organization [14,15]. In addition to the role of collagen in the maintenance of corneal transparency, the mechanical properties of the tissue are also dependent on the interactions of collagen molecules and the interactions of collagen with proteoglycans and water. Advanced structural models aimed at predicting the biomechanical response of the cornea to surgery and disease are therefore dependent on a detailed understanding of the three-dimensional organization of stromal collagen, swelling behaviour, and collagen–swelling interaction [16].

Corneal swelling studies, traditionally involving the direct immersion of corneas in distilled water and bathing solutions with different ionic strengths and pH levels, have enhanced understanding of the physiology of the cornea [17–19]. The discovery that direct immersion of the cornea in bathing solutions results in a significant loss of soluble proteins and proteoglycans [20] led to the development of an improved methodology for the *in vitro* manipulation of corneal hydration, in which the tissue is equilibrated to a given hydration using a bounding membrane that prevents the loss of proteoglycans [21]. This equilibration technique was successfully used in conjunction with X-ray scattering to examine the effect of pH and ionic strength on the swelling behaviour of the bovine corneal stroma [22].

X-ray scattering has been recognized as a highly sensitive tool for obtaining quantitative information about the structure of the cornea (averaged throughout the entire thickness of the tissue) in an unprocessed state, at both the molecular level and the fibrillar level [23,24]. Wide-angle X-ray scatter patterns from the cornea provide information about the average spacing between collagen molecules, while small-angle X-ray scatter patterns can be used to determine the diameter and separation distance of the fibrils. Using both small- and wide-angle X-ray scattering techniques, Meek *et al*. [21] showed that when the hydration of a dry bovine cornea is increased from *H* = 0 (*H*_dry_) to *H* = 1, intermolecular and interfibrillar spacing (IFS) increase in tandem, indicating that water entering the corneal stroma is distributed equally within and between the fibrils. However, as the hydration increases further (above *H* = 1), proportionally less water is absorbed into the fibrils. These findings were substantiated by Fratzl & Daxer's [25] X-ray investigations into the structural transformation of the human corneal stroma during air drying, in which they demonstrated that the fibrils themselves only become dehydrated when a critical point of drying (*H* = 1) is reached. The relationship between tissue hydration and IFS is very dependent on the pH and the ionic strength of the bathing medium [17,26]; thus, it is important to keep these parameters constant during swelling.

In this study, we have optimized the corneal equilibration technique to enable the accurate adjustment of stromal hydration in human, porcine, ovine and bovine corneas, thereby providing a means of returning posthumously swollen abattoir and eye bank tissue to physiological hydration. We use this technique to examine the molecular and fibrillar architecture of human and porcine corneas with small and wide-angle X-ray scattering at different equilibrated hydrations and also during the process of air drying, to quantify the relationship between corneal structure and water partitioning in each species.

## 2. Material and methods

### 2.1. Sample preparation

A total of 74 porcine eyes, 35 bovine eyes and 30 ovine eyes were obtained from a local abattoir. Following careful examination of each eye (to confirm the absence of epithelial erosion or stromal opacity), the corneal epithelium was debrided using a razor blade. The cornea was subsequently dissected from the eye and the endothelium gently removed. An 8 mm full-tissue thickness disc was trephined from the centre of each cornea (using a skin biopsy punch) and weighed. Nineteen porcine corneal discs were wrapped tightly in thin plastic catering film (Clingfilm™, Lidl, UK) and frozen at −80°C until required for X-ray data collection. The remaining porcine, bovine and ovine corneal discs were individually placed within 12–14 kD cut-off dialysis tubing. The tubing was carefully smoothed to remove any air bubbles and ensure close contact between the anterior and posterior surfaces of the stroma and the tubing. The tubing was then clamped on either side of the cornea with mediclips to form a tight seal. In this way, the close contact between the dialysis membrane and the cornea was maintained by surface tension throughout the de-swelling process. The sealed corneal discs were then immersed into solutions containing 5 mM HEPES buffer, 0.154 M sodium chloride and varying concentration polyethylene glycol (PEG) between 0 and 25% (PEG, Fluorochem Ltd, 20 kD MW) at pH 7.4 for the porcine corneas and 0–4% PEG for the ovine and bovine corneas. Following a preliminary investigation involving five bovine and five porcine corneas to assess the minimum time required for equilibration to occur (see the electronic supplementary material, S1 for details), equilibration was carried out at 4°C for 2 days for all remaining samples.

Sixty post-mortem human donor corneas aged between 46 and 87 years of age (average 71 ± 11 years) were obtained from Bristol Eye Bank following storage in culture media for a period of one to two months. After removal of the epithelial and endothelial cell layers, central corneal sections were cut out, placed in dialysis tubing and allowed to equilibrate at 4°C for 2 days in a PEG solution with a concentration of between 1 and 30%.

### 2.2. X-ray data collection and analysis

Thirty-five equilibrated human corneal samples were examined using small-angle X-ray scattering on Station 2.1, at the now decommissioned Daresbury Synchrotron Radiation Source (Warrington, UK). Twenty-five equilibrated human corneas, 50 equilibrated porcine corneas and three non-equilibrated porcine corneas were transported to Diamond Light Source (Didcot, UK) for examination on beamlines I22 (small-angle X-ray scattering) and I02 (wide-angle X-ray scattering). A further 16 non-equilibrated porcine corneas were transported to the European Synchrotron Radiation Source (ESRF, Grenoble, France) for examination on beamline ID-13 (wide-angle X-ray scattering).

Immediately prior to data collection, all corneas were brought to room temperature. The equilibrated corneal tissues were removed from the dialysis tubing, weighed, wrapped in Clingfilm to prevent tissue dehydration and placed in an air-tight sample holder. Small-angle X-ray scatter patterns were obtained from the centre of 26 equilibrated pig corneal discs and 14 equilibrated human corneal specimens using a 2–5 s exposure to a 0.1 nm wavelength X-ray beam. The X-ray scatter patterns were gathered on a detector positioned 6 m behind the sample and a lead beam stop was positioned between the sample and the detector to stop any un-deviated X-rays. In the case of the three non-equilibrated porcine corneas, small-angle X-ray scatter patterns were collected from the centre of each cornea using similar experimental parameters to those described above. The corneas were then left to air dry on the laboratory bench at 22°C for 5 min. Each corneal disc was then re-weighed, wrapped in cling film and used to generate another X-ray scatter pattern. This process was repeated until the change in weight became negligible.

On beamline I02 (Diamond Light Source), wide-angle X-ray scatter patterns were obtained from the centre of 24 equilibrated pig corneal discs and 11 equilibrated human corneal discs using a 0.5 s exposure to a 0.1 nm wavelength X-ray beam. The resulting X-ray scatter patterns were recorded on a detector positioned 30 cm behind the sample.

Further wide-angle X-ray scattering data were obtained from 16 non-equilibrated porcine corneas on beamline ID-13 (ESRF) using a 1 s exposure to a 0.1 nm wavelength X-ray beam. A number of X-ray scatter patterns were collected from the centre of each cornea during the process of air drying. In order to calculate corneal hydration, the wet weight of the corneal disc was recorded prior to the collection of each X-ray scatter pattern and a dry weight was obtained following 7 days storage in a 60°C oven.

**Figure 1.**
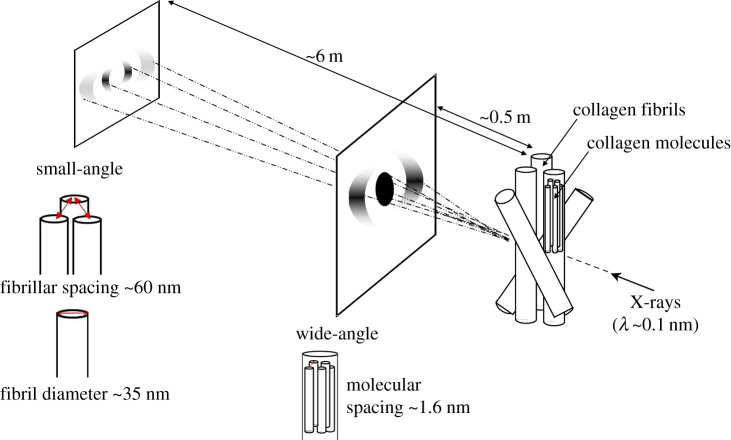
Schematic of corneal multi-modal X-ray scattering. X-rays are directed perpendicular to the corneal plane. The resulting X-ray scatter pattern is collected on a detector positioned behind the specimen. The distribution of small-angle X-ray scatter provides information about collagen parameters at the fibrillar level, whereas the wide-angle X-ray scatter provides information at the molecular level. (Online version in colour.)

**Figure 2.**
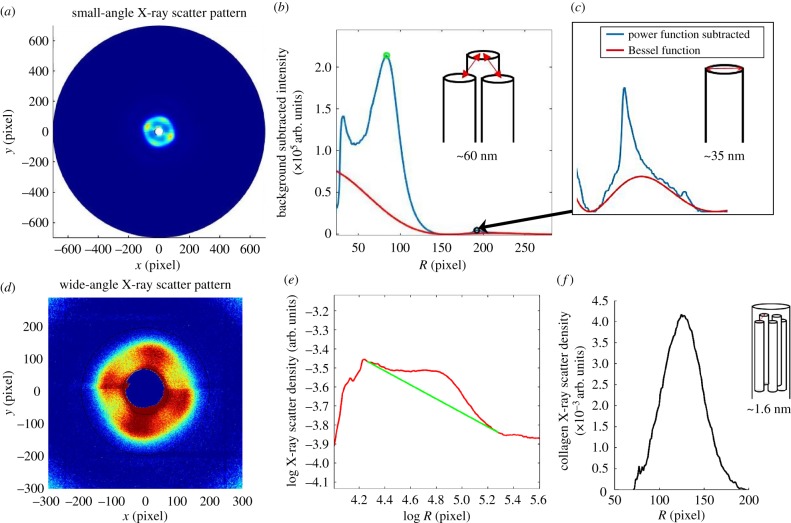
Analysis of X-ray scattering data. (*a*) The small-angle X-ray scatter pattern, which is circumferentially integrated to give a radial intensity profile. (*b*) Background-subtracted intensity profile (blue line), showing interference function peak (green circle) which provides a measure of the centre-to-centre separation distance of collagen fibrils. The fitted cylinder transform peak (Bessel function shown as a red line in *b* and inset *c*), which sits below the sharp third-order meridional reflection from corneal collagen, quantifies the average fibril diameter. (*d*) The wide-angle X-ray scatter pattern, which is circumferentially integrated around the collagen intermolecular reflection to give a radial intensity profile. (*e*) Radial intensity profile (red line), with fitted background function (green line). (*f*) The background-subtracted position of the intermolecular peak, once calibrated, gives a measure of the average collagen intermolecular separation.

Using Matlab software (Mathworks, USA), the small and wide-angle X-ray scattering data were analysed to obtain measurements of fibrillar and molecular collagen parameters as an average throughout the entire stromal thickness (figure 1). Analysis of small-angle X-ray scattering patterns has been described in detail previously [27]. Briefly, the background-subtracted and calibrated position of the interference function peak provides a measure of the average interfibrillar Bragg spacing (figure 2*a,b*), and the fitted cylinder transform peak can be used to quantify the average fibril diameter (figure 2*b,c*). The circumferentially integrated radial profile of the wide-angle X-ray scatter pattern gives rise to a collagen intermolecular peak (figure 2*d,e*). Once calibrated, the background-subtracted peak provides a measure of the collagen intermolecular Bragg spacing (figure 2*f*). The relationship between Bragg spacing and the corresponding centre-to-centre distance of the parameter under investigation depends on the precise packing of the molecules within the fibrils, or of the fibrils within the stroma. Most previous investigations have assumed a liquid-like packing [28,29], in which case Bragg spacings need to be multiplied by a factor of 1.1–1.2 in order to convert to centre-to-centre spacings. However, as we are only concerned here with changes in these parameters, we present all results as Bragg spacings.

### 2.3. Statistical analysis

Measurements of collagen fibril diameter, IFS and intermolecular spacing at specific levels of hydrations were statistically evaluated for human and porcine corneas using Student's *t*-tests to compare means. Student's *t*-test was also used to compare the slopes of the regression lines for collagen IFS versus hydration in human and pig corneas. The transition points described in the Results section, for both intermolecular spacing and fibril diameter, were determined by fitting a regression line to the data and determining the *R*^2^-value. The *R*^2^-value was monitored as data points were removed from the upper end of each graph (starting with all data and ending with the lowest three data points). The hydration at which the highest *R*^2^-value was obtained, indicating the best fit of the data to a line, was taken to mark the point of transition.

## 3. Results

### 3.1. Species study of the relationship between polyethylene glycol concentration and equilibrated stromal hydration

Following a minimum of one month storage in culture medium, the human corneas had an average hydration of *H* = 9 ± 1.8. The bovine and ovine corneas were obtained 18 h posthumously and had an initial hydration of *H* = 5.4 ± 0.6 and *H* = 4.7 ± 1.0, respectively. Porcine corneas were obtained within 4 h of death and remained closest to *H*_phys_ with a hydration of *H* = 3.0 ± 0.6.

**Figure 3.**
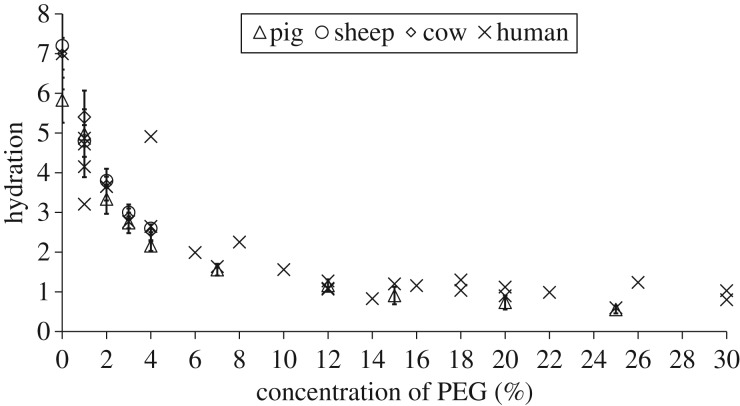
The relationship between the concentration of PEG in the bathing medium and the equilibrated stromal hydration in each species. Data for porcine, ovine and bovine corneas are shown as average values with standard deviation bars based on *n* = 5 at each data point. Data for human corneas represent a single specimen at each concentration. See the electronic supplementary material, S2 for tabulated data format.

Figure 3 shows the effect of different concentrations of PEG on the equilibrated stromal hydration of each species examined. Discounting the outlying data from one human cornea (4% PEG, *H* = 5), the relationship between PEG concentration and stromal hydration was seen to be similar for all species. However, the response of human donor corneas to equilibration in a given concentration of PEG was more variable than that of the other species examined. In each case, a hydration close to *H*_phys_ was achieved with a 2–3% PEG solution.

### 3.2. Structural transformation of the human and porcine corneal stroma with changing hydration

**Figure 4.**
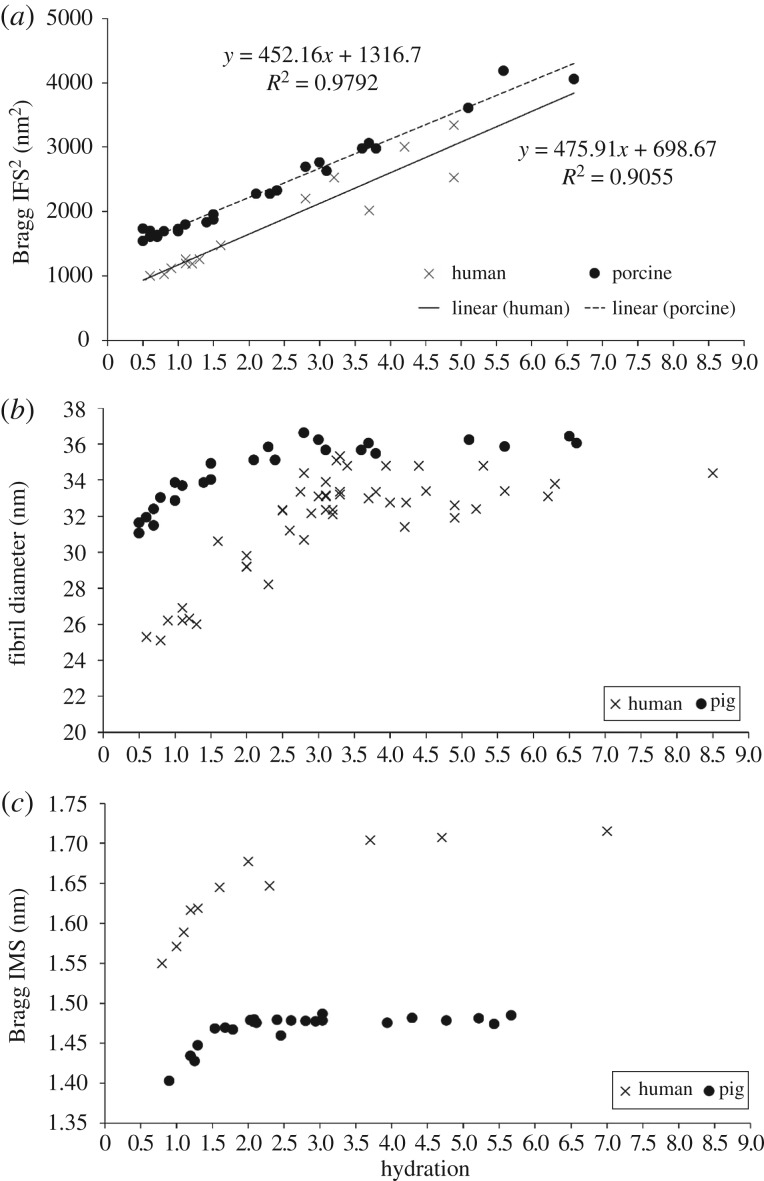
Structural transformation of the human and porcine corneal stroma with PEG adjusted hydration. The relationship between hydration and the square of the corneal collagen Bragg interfibrillar spacing (IFS^2^) (*a*), fibril diameter (*b*) and intermolecular spacing (IMS) (*c*) for PEG-equilibrated human and porcine corneas. See the electronic supplementary material, S3 for tabulated data format.

As IFS is known to expand and contract in two dimensions [17], figure 4*a* shows the square of the collagen IFS (IFS^2^) plotted as a function of stromal hydration. Both human and porcine corneal stroma exhibited a similar, strong positive linear relationship between IFS^2^ and hydration (figure 4*a*), with a stronger correlation (*R*^2^ = 0.98) in the pig corneas than in the human corneas (*R*^2^ = 0.91).

Fibril diameter and intermolecular spacing versus hydration obeyed a nonlinear, bi-phasic trend with both parameters showing an initial sharp, linear increase until a point where very little further increase occurs (figure 4*b,c*). However, this point of transition was seen to differ between intermolecular spacing and fibril diameter. While the intermolecular spacing levelled off at about *H* = 1.5 in both species, the diameter continued to increase before levelling off at *H* ≥ 3. Fibril diameter was significantly higher in pigs than in humans at all hydrations (*p* < 0.0001) (figure 4*b*), whereas the intermolecular spacing was significantly lower in pigs compared with human at all hydrations (*p* < 0.0001) (figure 4*c*).

### 3.3. Structural transformation of the porcine corneal stroma during air-drying versus equilibration

**Figure 5.**
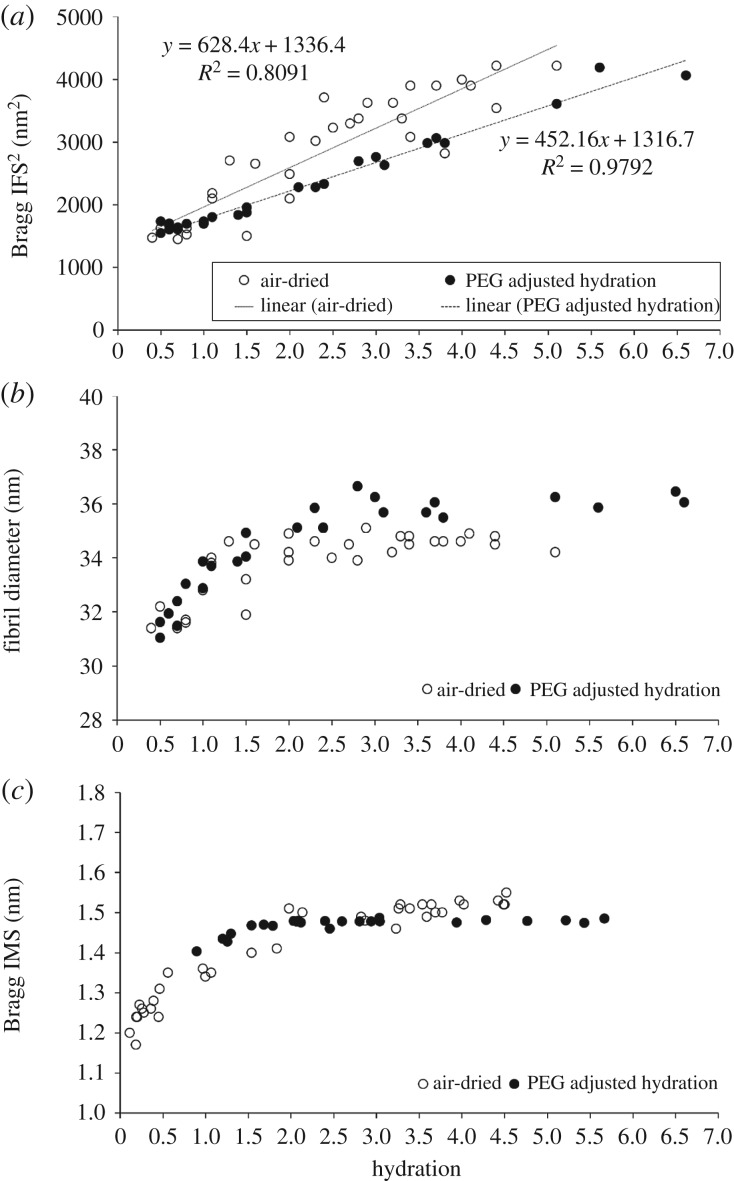
Structural transformation of the porcine corneal stroma during air drying and equilibration. The relationship between hydration and the square of the corneal collagen Bragg interfibrillar spacing (IFS^2^) (*a*), fibril diameter (*b*) and intermolecular spacing (IMS) (*c*) for air-dried corneas and PEG-equilibrated corneas. See electronic supplementary material S4 for tabulated data format.

As for PEG-equilibrated corneas, a positive linear correlation between IFS^2^ and hydration was observed in the non-equilibrated porcine corneas throughout the process of air-drying (figure 5*a*). Although, as with PEG equilibration, changes in fibril diameter occurred in a nonlinear, bi-phasic manner, the transition point seemed to shift from *H* = 3 to nearer *H* = 2.4, with a rapid decrease in diameter as hydration was reduced below this (figure 5*b*). Although there was no significant difference in the slope of the two regression lines shown in figure 5*a*, the PEG-equilibrated corneas showed an almost perfect correlation between IFS^2^ and hydration (*R*^2^ = 0.98) whereas the relationship was less predictable in the air-dried corneas (*R*^2^ = 0.80). Furthermore, analysis of fibril diameter measurements revealed that the PEG-equilibrated corneas had a significantly larger average fibril diameter than the air-dried corneas at all hydrations above *H* = 2.5 (*p* < 0.0001), but no difference in fibril diameter was detected at lower levels of hydration (figure 5*b*). The relationship between collagen intermolecular spacing and hydration appeared to be the same in both the equilibrated and air-dried corneas (figure 5*c*).

## 4. Discussion

In this study, we developed a protocol to control accurately the hydration of the corneal stroma of several species, and applied it in a detailed analysis of the hierarchical structural response of human and pig stroma to changes in hydration. Our quantification of water partitioning in these corneas at different tissue hydrations complements previous X-ray scattering studies examining the effect of hydration on stromal collagen parameters in PEG-equilibrated bovine corneas [21] and air-dried human corneas [25]. Furthermore, the numerical data we have obtained will enhance biomechanical models aimed at predicting the response of the human cornea to pathological conditions such as Fuch's corneal dystrophy, which involve significant stromal swelling [16].

The swelling of the corneal stroma is mostly governed by the Donnan osmotic pressure between the tissue (a polyelectrolyte containing negative fixed charge density) and its external solution. This pressure arises from the unequal distribution of small permeant ions between the two. Varying the concentration in the bathing medium of a dissolved molecule (such as PEG) that cannot enter the tissue, sets up an osmotic gradient between the inside and outside of the tissue that will draw water out of, or drive water into, the stroma. At equilibrium, this gradient will balance the stromal swelling pressure. Away from equilibrium, the cornea will swell or dehydrate in order to balance the osmotic gradient. As the cornea swells, the pressure difference, and hence the swelling pressure (*p*), reduces according to equation (4.1), where *c* is a constant [30].4.1
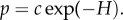


For a given fixed charge density in the sample, the swelling pressure, in Donnan theory, will be almost a linear function of the PEG concentration. Therefore, the higher the PEG concentration in the bathing solution, the lower the hydration. The majority of the fixed charge density on which the swelling pressure depends exists on the GAG side chains of the proteoglycans, although some resides in a chloride binding ligand [31].

Scott & Bosworth [32] have shown for several species that the polyanionic charge per unit volume is the same, so one would expect the constant *c* to be the same across species. It is therefore not surprising that we found that the concentration effect of PEG on tissue hydration is species independent. However, the predictability of stromal hydration adjustment based on PEG concentration was notably lower in the human donor corneas compared with the other species. The greater inter-specimen variability in the human corneas may be due to the loss of some proteoglycans during long-term storage in culture media [33,34], and/or the relative maturity of the human donor tissue (mean age of 71 years) and associated changes in proteoglycan composition that occur with age [35].

**Figure 6.**
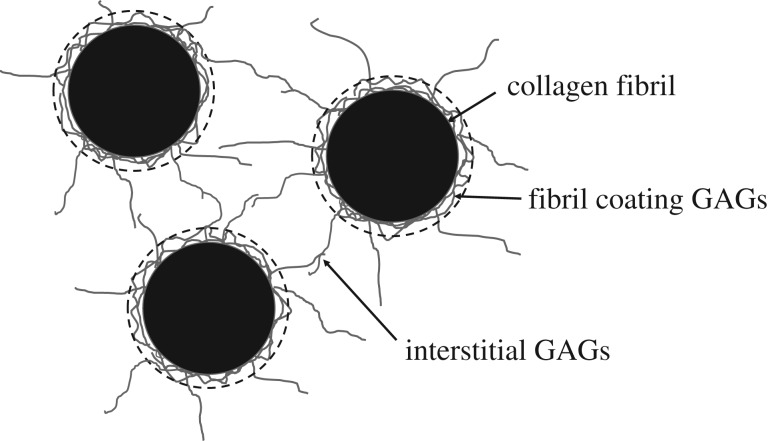
Schematic of three collagen fibrils in cross-section (black) with associated glycosaminoglycans (GAGs; grey). For the purposes of this paper, the GAGs have been divided into two components: fibril coating GAGs and interstitial GAGs. X-ray scattering measurements of fibril diameter include the cylindrical collagen fibril plus the surrounding fibril coating (shown as a broken black line).

In this study, we also noticed several important differences in the swelling/drying response of different species, which together give a detailed picture of how fixed charge density is likely to be distributed in each tissue. As is known to be the case for PEG-equilibrated bovine corneas [21] and air-dried human corneas [25], the current data show that a positive linear correlation also exists between stromal hydration and IFS^2^ in equilibrated human and porcine corneas and air-dried porcine corneas. Meek *et al*. [21] showed in bovine cornea that, as water enters the stroma from very low hydrations, it goes equally within and between the fibril, up to about *H* = 1. Above physiological hydration, the fibrils themselves swell very little, with all the extra water entering the extrafibrillar space and thus moving the fibrils apart. Fratzl & Daxer [25] used these data in combination with measurements from human corneas to postulate that the collagen fibrils are surrounded by a ‘fractal’ coating, consisting of proteoglycan core proteins and associated GAGs, which maintains a separation distance between fibrils even when dry but does not contribute to X-ray measurements of fibril diameters. They estimated that the diameter of the dry fibril cores was 26 nm in humans, and that the fractal coating surrounding them was about 5.25 nm thick. Cheng & Pinsky [36] later proposed a more detailed model of the collagen/GAG arrangement, in which the GAGs were divided into two components: fibril coating GAGs and interstitial GAGs (figure 6). The fibril coating GAGs prevent fibrils from getting so close that they are able to fuse together [37], while the interstitial GAGs provide a restoring force that helps to maintain fibrils in their ordered lattice arrangement [36,38].

**Figure 7.**
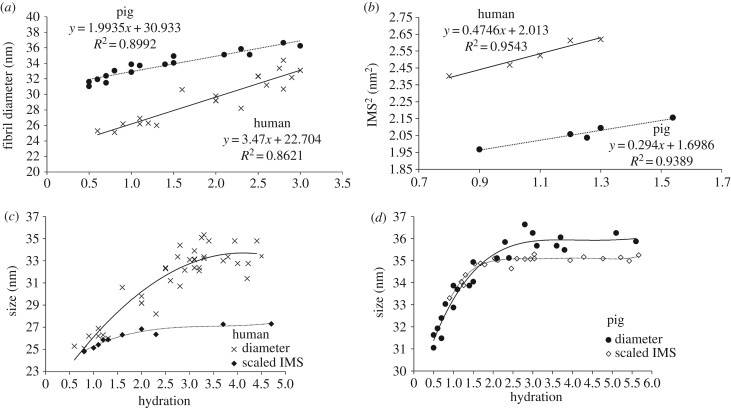
Fibril diameter data (from figure 4) and intermolecular spacing^2^ (IMS^2^) are shown at low hydrations, where the behaviour is linear. Intermolecular spacings are normalized to match the value of the corresponding fibril diameter at *H* = 0 for human (*c*) and pig (*d*) corneas, based upon the assumption that the thickness of the GAG coating is negligible when the fibrils are dry. A best-fit polynomial has been applied to each dataset.

Contrary to Fratzl & Daxer [25] and based on our current findings, we propose that fibril diameters measured by X-ray diffraction include the collagen fibril core together with a contribution from the fibril coating. This is plausible based on evidence that proteoglycans can scatter X-rays [24,39]. Between *H* = 3 and *H* = 0.5, there is good linear relationship between diameter and hydration from which it is possible to estimate the dry fibril diameters (including the coating). For human, this value is approximately 23 nm and for pig, approximately 31 nm (figure 7*a*). These values are in close accord with electron microscopy measurements of fibril diameter obtained from human [40] and pig corneas [41], which are presumed to be in their fully dehydrated state. The observed species difference in fibril diameter may be explained by the presence of a greater number of molecules in the pig collagen fibril cross-section than in the human, as previously suggested by Meek & Leonard [42].

From figure 4*b*, it can be seen that for both human and pig, the diameter does not change above physiological hydration. This implies that molecules are separated to the maximum extent permitted by their intermolecular cross-links, and that the fibril coating is fully water-saturated and cannot therefore expand. Below *H* = 3, water is lost initially from the fibril coating and then from the collagen fibrils themselves. The point at which water is lost from the fibrils can be seen in figure 4*c*, where the intermolecular spacing starts to reduce (*H* < 1.5). This is slightly more than the values quoted by Meek *et al*. [21] and Fratzl & Daxer [25], but in both of these cases, hydration was measured by vacuum drying, whereas here it was measured by oven drying, which is known to lead to higher values for *H* [2].

The partitioning of water in connective tissues is a function of the osmotic pressure difference between the extrafibrillar and intrafibrillar spaces [43]. The fixed charge density that gives rise to this osmotic pressure difference largely resides within the proteoglycans, particularly those that coat the outside of the collagen fibrils [44]. Figure 7*b* shows intermolecular spacing^2^ versus hydration at low hydrations. Linear trends are observed because, as for the IFS, the molecules expand or contract in two dimensions. The intermolecular spacing is consistently higher in the human than in the pig, indicating that at any given tissue hydration, there is a greater osmotic differential between the inside and outside of the fibrils in the pig cornea. The fibrils in the pig cornea will thus reach equilibrium with their surroundings with a lower fibril water content, drawing the molecules closer together. A species difference in the osmotic differential may be due to a higher fixed charge density in pig fibril coating GAGs. It may also be due to a diminished fixed charge density in the human corneas following long-term storage in organ culture caused by the loss of some fibril coating proteoglycans and/or the presence of a third proteoglycan component, which has been purported to exist inside the human corneal collagen fibrils [45].

By extrapolation to *H* = 0, we find that the closest approach of the molecules is 1.30 nm in the pig and 1.42 nm in the human. The larger intermolecular spacing in the dry human cornea probably reflects a higher degree of cross-linking. Differences in the number and strength of these covalent bonds may be due to an age effect caused by our use of relatively young pig corneas (less than 1 year of age and roughly equivalent to 18 human years) and more mature human corneas (mean age of 71 years). X-ray scattering studies have previously demonstrated that human corneal collagen fibril diameter increases with age as a result of a glycation-induced expansion of collagen intermolecular spacing [46] and the incorporation of additional collagen molecules [47]. This natural variation could contribute to the high level of scatter in the human data in figure 4*b* compared with the pig.

In figure 7*c,d*, the intermolecular spacings from figure 4*c* have been normalized to match the value of the corresponding fibril diameters at *H* = 0, on the assumption that the thickness of the GAG fibril coating is negligible when the fibrils are dry, the geometry of the molecular arrangement within the fibril core is not altered during drying and the linear relationship seen between hydration and both intermolecular spacing and fibril diameter at low hydrations, is maintained until *H* = 0. Normalization was carried out by dividing the dry fibril diameter by the dry intermolecular spacing to give a value that is proportional to the number of molecular spacings across the fibril diameter. This number is a constant, as the total number of molecules in the fibril is independent of hydration. So, by multiplying the intermolecular spacing measured at a given hydration by this normalization constant, we can predict how the diameter of the fibril core alone would be expected to change with increasing hydration. The lower plot in figure 7*c,d* represents the behaviour of the collagen fibrils as the molecules move apart during swelling, whereas the higher plot in each graph represents the diameter including the fibril coating (the measured fibril diameter data in figure 4*b*). The difference therefore represents the change in the thickness of the coating as fibrils swell. From this, it can be seen that, when hydrated (*H* > 3), the diameter including the GAG coating is about 5 nm thicker than the collagen fibril (coating thickness about 2.5 nm) for the human, and about 1 nm thicker (coating thickness about 0.5 nm) in pig. Above physiological hydration, the coating thickness seems to remain constant, suggesting that the coating GAGs have saturated. The reason for this is unclear, although it could be because the types of proteoglycan in the coating are less osmotically active than those elsewhere and therefore saturate sooner [38,48,49].

Detailed investigation into the structural transformation of equilibrated and air-dried pig corneas with changing hydration revealed small but significant differences between the two hydration adjustment techniques, with the PEG-equilibrated corneas showing a larger fibril diameter than the air-dried corneas between *H* = 2.5 and 4. As no difference in the intermolecular spacing was observed within this hydration range, the implication is that the behaviour of the fibril coating GAG component differs depending on the method of hydration adjustment used. This is not wholly surprising as the equilibration technique allows the diffusion of ions backwards and forwards across a semi-permeable membrane while air-drying will produce an increased concentration of chloride ions within the stroma, a situation which is known to affect the structural organization of the tissue [50].

Furthermore, the air-drying method is likely to produce a non-uniform change in hydration across the cornea, with greater drying occurring at the anterior and posterior surfaces than within. As each X-ray scatter pattern represents an average measurement throughout the entire tissue thickness, the relationship between corneal hydration and the modal average measurement for each collagen parameter would therefore be expected to be less precise than achieved with the equilibration method which adjusts hydration consistently throughout the stroma. While the air-drying technique offers a quick and drastic adjustment of corneal hydration, we have demonstrated that the equilibration method provides a slower, more controlled means of hydration adjustment, via a mechanism that is closer to the physiological behaviour of the tissue.

In conclusion, we have shown that species variations exist in the structural response of the cornea to hydration that can be explained by differences in molecular cross-linking and intra/interfibrillar water partitioning. While overall the total fixed charge density within the tissue is the same in all species, human corneal collagen fibrils appear to have a thicker GAG coating with a lower fixed charge density than found in the pig. This lower fixed charge density means that the human collagen fibrils are more hydrated than those in the pig (at a given overall tissue hydration), as water partitioning between the inside and outside of the fibril is governed by the osmotic pressure gradient between the extrafibrillar and intrafibrillar matrix.

## Supplementary Material

Time required for equilibration to be achieved

## Supplementary Material

Species study of the relationship between PEG concentration and equilibrated stromal hydration

## Supplementary Material

Structural transformation of the human and porcine corneal stroma with changing hydration

## Supplementary Material

Structural transformation of the porcine corneal stroma during air-drying versus equilibration

## Data accessibility

All of the data presented in this paper are provided in tabulated format in the electronic supplementary material.
