# Supplementary material for: The structural response of the cornea to changes in stromal hydration
Source: J R Soc Interface. 2017 Jun 7;14(131):20170062. doi: 10.1098/rsif.2017.0062 (PMC5493790; doi:10.1098/rsif.2017.0062)
Supplement: Time required for equilibration to be achieved [file rsif20170062supp1.doc]

**S1. Time required for equilibration to be achieved**

*Sample preparation and methods*

Five bovine and five porcine corneas were used to investigate the time required for corneal equilibration to occur in a 4% PEG solution. At 3 hr, 5 hr, 24 hr, 28 hr and 48 hrs the corneal discs were removed from their dialysis tubing and weighed. After 48 hr the corneal disks were placed in a 60 ºC oven and dried for 3 days until a constant dry weight was achieved. The hydration at each time point during the equilibration process was then calculated.

*Results*

The bovine and porcine corneas used in this part of the study were obtained within 18 hrs of death and had an initial hydration of H = 6.1 ± 0.8 and H = 6.1 ± 0.7 respectively. After 28 hours in a 4% PEG solution, equilibration was achieved in both the bovine and porcine corneal stroma (Figure 1).


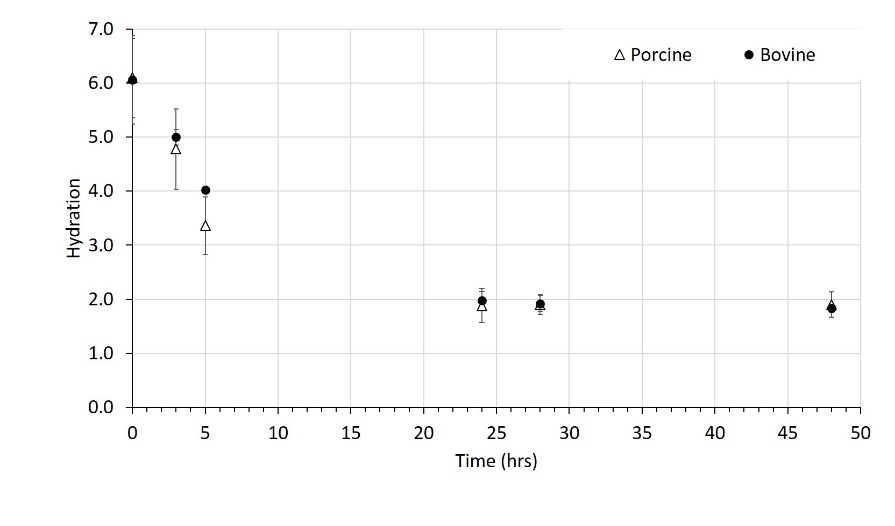


Figure 1: Measurement of bovine and porcine stromal hydration with time in a 4% PEG bathing medium.
