# Supplementary material for: The structural response of the cornea to changes in stromal hydration
Source: J R Soc Interface. 2017 Jun 7;14(131):20170062. doi: 10.1098/rsif.2017.0062 (PMC5493790; doi:10.1098/rsif.2017.0062)
Supplement: Species study of the relationship between PEG concentration and equilibrated stromal hydration [file rsif20170062supp2.doc]

**S2: Species study of the relationship between PEG concentration and equilibrated stromal hydration**

Tabulated format of data presented in Figure 3.

| **PEG concentration (%)** | **pig** | **human** | **sheep** | **cow** |
| --- | --- | --- | --- | --- |
| 0 | 5.8 ± 0.6 (n = 6) | 7.0  (n = 1) | 7.2 ± 1.1 (n = 6) | 7.0 ± 0.4 (n = 6) |
| 1 | 5.0 ± 1.1 (n = 6) | 4.2 ± 0.8  (n = 4) | 4.8 ± 0.4 (n = 6) | 5.4 ± 0.2 (n = 6) |
| 2 | 3.3 ± 0.4 (n = 6) | 3.7, 3.7  (n = 2) | 3.8 ± 0.1 (n = 6) | 3.7 ± 0.4 (n = 6) |
| 3 | 2.8 ± 0.2 (n = 7) | 2.8  (n = 1) | 3.0 ± 0.1 (n = 6) | 2.9 ± 0.3 (n = 6) |
| 4 | 2.2 ± 0.1 (n = 7) | 2.6, 4.9  (n = 2) | 2.6 ± 0.1 (n = 6) | 2.5 ± 0.2 (n = 6) |
| 6 |  | 2.0  (n = 1) |  |  |
| 7 | 1.6 ± 0.1 (n = 6) | 1.6  (n = 1) |  |  |
| 8 |  | 2.3  (n = 1) |  |  |
| 10 |  | 1.6  (n = 1) |  |  |
| 12 | 1.2 ± 0.2 (n = 4) | 1.2 ± 0.2  (n = 3) |  |  |
| 14 |  | 0.8  (n = 1) |  |  |
| 15 | 0.9 ± 0.2 (n = 4) | 1.2  (n = 1) |  |  |
| 16 |  | 1.2  (n = 1) |  |  |
| 18 |  | 1.0, 1.3  (n = 2) |  |  |
| 20 | 0.7 ± 0.2 (n = 3) | 1.1 ± 0.1  (n = 3) |  |  |
| 22 |  | 1.0  (n = 1) |  |  |
| 25 | 0.6 ± 0.1 (n = 3) | 0.6  (n = 1) |  |  |
| 26 |  | 1.2  (n = 1) |  |  |
| 30 |  | 0.8, 1.0  (n = 2) |  |  |
