# Supplementary material for: The structural response of the cornea to changes in stromal hydration
Source: J R Soc Interface. 2017 Jun 7;14(131):20170062. doi: 10.1098/rsif.2017.0062 (PMC5493790; doi:10.1098/rsif.2017.0062)
Supplement: Structural transformation of the human and porcine corneal stroma with changing hydration [file rsif20170062supp3.doc]

**S3: Structural transformation of the human and porcine corneal stroma with changing hydration**

Tabulated format of data presented in Figure 4A.

| **HUMAN** | | **PIG** | |
| --- | --- | --- | --- |
| **Hydration** | **Bragg interfibrillar spacing² (nm²)** | **Hydration** | **Bragg interfibrillar spacing² (nm²)** |
| 0.8 | 1024.0 | 0.5 | 1733.1 |
| 0.6 | 1004.9 | 0.5 | 1544.5 |
| 0.9 | 1122.3 | 0.6 | 1699.1 |
| 1.3 | 1260.3 | 0.6 | 1603.2 |
| 1.2 | 1190.3 | 0.7 | 1603.2 |
| 1.1 | 1260.3 | 0.7 | 1634.6 |
| 1.1 | 1197.2 | 0.8 | 1694.1 |
| 1.6 | 1474.6 | 1 | 1728.1 |
| 4.9 | 2530.1 | 1 | 1694.1 |
| 2.8 | 2199.6 | 1.1 | 1798.6 |
| 3.7 | 2016.0 | 1.4 | 1835.3 |
| 3.2 | 2530.1 | 1.5 | 1952.8 |
| 4.2 | 3003.0 | 1.5 | 1873.2 |
| 4.9 | 3340.8 | 2.1 | 2276.2 |
|  |  | 2.3 | 2276.2 |
|  |  | 2.4 | 2329.0 |
|  |  | 2.8 | 2694.6 |
|  |  | 3 | 2762.6 |
|  |  | 3.1 | 2629.6 |
|  |  | 3.6 | 2982.3 |
|  |  | 3.7 | 3060.3 |
|  |  | 3.8 | 2982.3 |
|  |  | 5.1 | 3608.4 |
|  |  | 5.6 | 4184.8 |
|  |  | 6.6 | 4059.0 |

Tabulated format of data presented in Figure 4B.

|  | **HUMAN** |  | **PIG** |
| --- | --- | --- | --- |
| **Hydration** | **Fibril diameter (nm)** | **Hydration** | **Fibril diameter (nm)** |
| 0.6 | 25.3 | 0.5 | 31.6 |
| 0.8 | 25.1 | 0.5 | 31.0 |
| 0.9 | 26.2 | 0.6 | 31.9 |
| 1.1 | 26.9 | 0.6 | 31.9 |
| 1.1 | 26.2 | 0.7 | 32.4 |
| 1.2 | 26.3 | 0.7 | 31.5 |
| 1.3 | 26.0 | 0.8 | 33.0 |
| 1.6 | 30.6 | 1.0 | 33.9 |
| 2.0 | 29.8 | 1.0 | 32.9 |
| 2.0 | 29.2 | 1.1 | 33.7 |
| 2.0 | 29.2 | 1.4 | 33.9 |
| 2.3 | 28.2 | 1.5 | 34.9 |
| 2.5 | 32.4 | 1.5 | 34.0 |
| 2.5 | 32.3 | 2.1 | 35.1 |
| 2.6 | 31.2 | 2.3 | 35.8 |
| 2.8 | 33.4 | 2.4 | 35.1 |
| 2.8 | 34.4 | 2.8 | 36.6 |
| 2.8 | 30.7 | 3.0 | 36.2 |
| 2.9 | 32.2 | 3.1 | 35.7 |
| 3.0 | 33.1 | 3.6 | 35.7 |
| 3.0 | 33.1 | 3.7 | 36.1 |
| 3.1 | 33.9 | 3.8 | 35.5 |
| 3.1 | 33.1 | 5.1 | 36.2 |
| 3.1 | 33.2 | 5.6 | 35.9 |
| 3.1 | 32.4 | 6.5 | 36.4 |
| 3.1 | 32.4 | 6.6 | 36.1 |
| 3.2 | 32.1 |  |  |
| 3.2 | 32.4 |  |  |
| 3.3 | 35.1 |  |  |
| 3.3 | 33.2 |  |  |
| 3.3 | 35.3 |  |  |
| 3.3 | 33.4 |  |  |
| 3.4 | 34.8 |  |  |
| 3.7 | 33.0 |  |  |
| 3.8 | 33.4 |  |  |
| 3.9 | 34.8 |  |  |
| 4.0 | 32.8 |  |  |
| 4.2 | 31.4 |  |  |
| 4.2 | 32.8 |  |  |
| 4.4 | 34.8 |  |  |
| 4.5 | 33.4 |  |  |
| 4.9 | 32.6 |  |  |
| 4.9 | 31.9 |  |  |
| 5.2 | 32.4 |  |  |
| 5.3 | 34.8 |  |  |
| 5.6 | 33.4 |  |  |
| 6.2 | 33.1 |  |  |
| 6.3 | 33.8 |  |  |
| 8.5 | 34.4 |  |  |

Tabulated format of data presented in Figure 4C.

|  | **HUMAN** |  | **PIG** |
| --- | --- | --- | --- |
| **Hydration** | **Bragg intermolecular spacing (nm)** | **Hydration** | **Bragg intermolecular spacing (nm)** |
| 0.8 | 1.55 | 0.9 | 1.40 |
| 1.0 | 1.57 | 1.2 | 1.43 |
| 1.1 | 1.59 | 1.3 | 1.43 |
| 1.2 | 1.62 | 1.3 | 1.45 |
| 1.3 | 1.62 | 1.5 | 1.47 |
| 1.6 | 1.64 | 1.7 | 1.47 |
| 2.0 | 1.68 | 1.8 | 1.47 |
| 2.3 | 1.65 | 2.0 | 1.48 |
| 3.7 | 1.70 | 2.1 | 1.48 |
| 4.7 | 1.71 | 2.1 | 1.48 |
| 7.0 | 1.72 | 2.1 | 1.48 |
|  |  | 2.4 | 1.48 |
|  |  | 2.5 | 1.46 |
|  |  | 2.6 | 1.48 |
|  |  | 2.8 | 1.48 |
|  |  | 2.9 | 1.48 |
|  |  | 3.0 | 1.49 |
|  |  | 3.0 | 1.48 |
|  |  | 3.9 | 1.48 |
|  |  | 4.3 | 1.48 |
|  |  | 4.8 | 1.48 |
|  |  | 5.2 | 1.48 |
|  |  | 5.4 | 1.47 |
|  |  | 5.7 | 1.48 |
