# Supplementary material for: The structural response of the cornea to changes in stromal hydration
Source: J R Soc Interface. 2017 Jun 7;14(131):20170062. doi: 10.1098/rsif.2017.0062 (PMC5493790; doi:10.1098/rsif.2017.0062)
Supplement: Structural transformation of the porcine corneal stroma during air-drying versus equilibration [file rsif20170062supp4.doc]

**S4: Structural transformation of the porcine corneal stroma during air-drying versus equilibration**

Tabulated format of data presented in Figure 5A.

|  | **PIG (equilibrated)** |  | **PIG (air dried)** |
| --- | --- | --- | --- |
| **Hydration** | **Bragg interfibrillar spacing² (nm²)** | **Hydration** | **Bragg interfibrillar spacing² (nm²)** |
| 0.5 | 1733.1 | 0.4 | 1474.6 |
| 0.5 | 1544.5 | 0.5 | 1624.1 |
| 0.6 | 1699.1 | 0.7 | 1451.6 |
| 0.6 | 1603.2 | 0.8 | 1624.1 |
| 0.7 | 1603.2 | 0.8 | 1521.0 |
| 0.7 | 1634.6 | 1.0 | 1730.6 |
| 0.8 | 1694.1 | 1.1 | 2180.9 |
| 1.0 | 1728.1 | 1.1 | 2097.6 |
| 1.0 | 1694.1 | 1.3 | 2704.0 |
| 1.1 | 1798.6 | 1.5 | 1953.6 |
| 1.4 | 1835.3 | 1.5 | 1497.7 |
| 1.5 | 1952.8 | 1.6 | 2652.3 |
| 1.5 | 1873.2 | 2.0 | 3080.3 |
| 2.1 | 2276.2 | 2.0 | 2490.0 |
| 2.3 | 2276.2 | 2.0 | 2097.6 |
| 2.4 | 2329.0 | 2.3 | 3014.0 |
| 2.8 | 2694.6 | 2.4 | 3708.8 |
| 3.0 | 2762.6 | 2.5 | 3226.2 |
| 3.1 | 2629.6 | 2.7 | 3294.8 |
| 3.6 | 2982.3 | 2.8 | 3375.6 |
| 3.7 | 3060.3 | 2.9 | 3624.0 |
| 3.8 | 2982.3 | 3.2 | 3624.0 |
| 5.1 | 3608.4 | 3.3 | 3375.6 |
| 5.6 | 4184.8 | 3.4 | 3080.3 |
| 6.6 | 4059.0 | 3.4 | 3893.8 |
|  |  | 3.7 | 3893.8 |
|  |  | 3.8 | 2819.6 |
|  |  | 4 | 3994.2 |
|  |  | 4.1 | 3893.8 |
|  |  | 4.4 | 3540.3 |
|  |  | 4.4 | 4212.0 |
|  |  | 5.1 | 4212.0 |

Tabulated format of data presented in Figure 5B.

|  | **PIG (equilibrated)** |  | **PIG (air dried)** |
| --- | --- | --- | --- |
| **Hydration** | **Fibril diameter (nm)** | **Hydration** | **Fibril diameter (nm)** |
| 0.5 | 31.6 | 0.4 | 31.4 |
| 0.5 | 31.0 | 0.5 | 32.2 |
| 0.6 | 31.9 | 0.7 | 31.4 |
| 0.6 | 31.9 | 0.8 | 31.6 |
| 0.7 | 32.4 | 0.8 | 31.7 |
| 0.7 | 31.5 | 1.0 | 32.8 |
| 0.8 | 33.0 | 1.1 | 34 |
| 1.0 | 33.9 | 1.1 | 33.8 |
| 1.0 | 32.9 | 1.3 | 34.6 |
| 1.1 | 33.7 | 1.5 | 33.2 |
| 1.4 | 33.9 | 1.5 | 31.9 |
| 1.5 | 34.9 | 1.6 | 34.5 |
| 1.5 | 34.0 | 2.0 | 34.2 |
| 2.1 | 35.1 | 2.0 | 34.9 |
| 2.3 | 35.8 | 2.0 | 33.9 |
| 2.4 | 35.1 | 2.3 | 34.6 |
| 2.8 | 36.6 | 2.4 | 35.1 |
| 3.0 | 36.2 | 2.5 | 34 |
| 3.1 | 35.7 | 2.7 | 34.5 |
| 3.6 | 35.7 | 2.8 | 33.9 |
| 3.7 | 36.1 | 2.9 | 35.1 |
| 3.8 | 35.5 | 3.2 | 34.2 |
| 5.1 | 36.2 | 3.3 | 34.8 |
| 5.6 | 35.9 | 3.4 | 34.5 |
| 6.5 | 36.4 | 3.4 | 34.8 |
| 6.6 | 36.1 | 3.7 | 34.6 |
|  |  | 3.8 | 34.6 |
|  |  | 4.0 | 34.6 |
|  |  | 4.1 | 34.9 |
|  |  | 4.4 | 34.5 |
|  |  | 4.4 | 34.8 |
|  |  | 5.1 | 34.2 |

Tabulated format of data presented in Figure 5C.

|  | **PIG (equilibrated)** |  | **PIG (air dried)** |
| --- | --- | --- | --- |
| **Hydration** | **Bragg intermolecular spacing (nm)** | **Hydration** | **Bragg intermolecular spacing (nm)** |
| 0.9 | 1.40 | 0.1 | 1.20 |
| 1.2 | 1.43 | 0.2 | 1.17 |
| 1.3 | 1.43 | 0.2 | 1.24 |
| 1.3 | 1.45 | 0.2 | 1.24 |
| 1.5 | 1.47 | 0.2 | 1.27 |
| 1.7 | 1.47 | 0.3 | 1.26 |
| 1.8 | 1.47 | 0.3 | 1.25 |
| 2.0 | 1.48 | 0.4 | 1.26 |
| 2.1 | 1.48 | 0.4 | 1.28 |
| 2.1 | 1.48 | 0.5 | 1.24 |
| 2.1 | 1.48 | 0.5 | 1.31 |
| 2.4 | 1.48 | 0.6 | 1.35 |
| 2.5 | 1.46 | 1.0 | 1.36 |
| 2.6 | 1.48 | 1.0 | 1.34 |
| 2.8 | 1.48 | 1.1 | 1.35 |
| 2.9 | 1.48 | 1.5 | 1.40 |
| 3.0 | 1.49 | 1.8 | 1.41 |
| 3.0 | 1.48 | 2.0 | 1.51 |
| 3.9 | 1.48 | 2.1 | 1.50 |
| 4.3 | 1.48 | 2.8 | 1.49 |
| 4.8 | 1.48 | 2.9 | 1.48 |
| 5.2 | 1.48 | 3.2 | 1.46 |
| 5.4 | 1.47 | 3.3 | 1.51 |
| 5.7 | 1.48 | 3.3 | 1.52 |
|  |  | 3.4 | 1.51 |
|  |  | 3.5 | 1.52 |
|  |  | 3.6 | 1.49 |
|  |  | 3.6 | 1.52 |
|  |  | 3.7 | 1.50 |
|  |  | 3.8 | 1.50 |
|  |  | 4.0 | 1.53 |
|  |  | 4.0 | 1.52 |
|  |  | 4.4 | 1.53 |
|  |  | 4.5 | 1.52 |
|  |  | 4.5 | 1.52 |
|  |  | 4.5 | 1.55 |
